# Cold atmospheric plasma in orthopaedic and urologic tumor therapy

**DOI:** 10.3205/dgkh000295

**Published:** 2017-08-08

**Authors:** Denis Gümbel, Georg Daeschlein, Axel Ekkernkamp, Axel Kramer, Matthias B. Stope

**Affiliations:** 1 Department of Trauma, Reconstructive Surgery and Rehabilitation Medicine, University Medicine Greifswald, Germany; 2 Department of Trauma and Orthopaedic Surgery, BG Klinikum Unfallkrankenhaus Berlin gGmbH, Berlin, Germany; 3 Department of Dermatology, University Medicine Greifswald, Germany; 4 Institute for Hygiene and Environmental Medicine, University Medicine Greifswald, Germany; 5 Department of Urology, University Medicine Greifswald, Germany

**Keywords:** cold atmospheric plasma, oncology, osteosarcoma, bladder cancer, prostate cancer

## Abstract

Cold atmospheric plasma (CAP) is a highly reactive ionized physical state thereby provoking divers biological effects. In medical applications, CAP treatment promotes wound healing, provokes immunostimulation, and is antiseptically active. Moreover, CAP interacts with antiproliferative mechanisms suggesting CAP treatment as a promising anticancer strategy. Here we review the current state of science concerning the so far investigated CAP effects on different cancer entities in orthopaedic and urologic oncology.

## Zusammenfassung

Kalte atmosphärische Plasmen (CAP) induzieren auf Grund ihres hoch reaktiven ionisierten Zustands eine Reihe biologischer Wirkungen wie Immunstimulation, Förderung der Wundheilung und Antisepsis. Außerdem werden durch CAP apoptotische Prozesse induziert und Zellregulationen carcinomatös entarteter Zellen mit dem Ergebnis einer antiproliferativen Wirkung beeinflusst, so dass der Einsatz von CAP im Rahmen eines multimodalen Konzepts zur Krebsbehandlung aussichtsreich erscheint. Im vorliegenden Minireview wird der Erkenntnisstand zur Wirkung von CAP auf orthopädische und urologische Krebserkrankungen analysiert.

## Introduction

Physical plasma is defined as a highly reactive ionized physical state containing diverse biologically reactive factors including charged particles, free radicals, excited atoms and molecules, photons, and electromagnetic fields. Advances in physics have enabled the use of pulsed physical atmospheric plasma (cold atmospheric plasma: CAP) for medical purposes, which operate by low pressure and at temperatures between 36°C and 52°C. In the beginning, CAP treatment of biological surfaces, e.g. in skin pathologies and dental diseases, took center stage. Here, primarily CAP-induced anti-microbial and immunostimulating effects have demonstrated beneficial effects for medical applications [1], [2], [3], [4], [5], [6], [7].

In surgery, and particularly in oncological surgery, the preservation of adjacent tissues and the protection of neighboring structures and organs is an important objective. An oncologically required degree of resection, however, is frequently prevented due to neighboring structures, such as blood vessels and nerves. Because of its anti-neoplastic properties [8], [9], [10], [11], [12], [13], [14], [15] as well as its quality to facilitate wound healing [16], [17], CAP application in oncology has increasingly moved into the focus of interest, constituting a novel field in plasma medicine: plasma oncology. Intraoperative CAP treatment of patients undergoing resection may inactivate cancer cells adjacent to critical sites and, moreover, may promote subsequent healing due to CAP’s antimicrobial and immunostimulating efficacy.

In the field of oncology only little is known about biological CAP effects and possible applications for anticancer therapy. An important advantage is that plasma can act selectively against cancer cells [18]. Also CAP-stimulated solutions and culture medium inactivate cancer cells *in vitro* with specific vulnerability of pancreatic adenocarcinoma cells and glioblastoma cells [19]. One reason is the higher susceptibility of cancer cells to CAP-induced reactive oxygen and nitrogen species (RONS; especially nitric oxide (NO) and nitrogen dioxide (NO^2–^) radicals) than normal cells, and consequently, CAP induces apoptotic cell responses primarily in cancer cells [14]. Moreover, CAP enhances cancer cell death *in vitro* by mitochondria-mediated apoptosis [20]. The CAP-induced apoptosis has been observed together with an accumulation of cells in S phase of the cell cycle, which suggests an arrest of tumor proliferation [21].

Here we review the current state of science concerning the so far investigated CAP effects on different cancer entities in orthopaedic and urologic oncology.

## Osteosarcoma

Diagnosis of osteosarcoma is routinely evaluated by histochemistry and is followed by a multimodal treatment including wide surgical resection of tumor tissue and multi-agent chemotherapy [22]. Recent *in vitro* data demonstrating the anticancer capability of CAP treatment in cell culture approaches might represent a promising option for the intraoperative inactivation of osteosarcoma cells.

Two osteosarcoma cell lines (U2-OS cells, MNNG/HOS cells) reflecting two different molecular subtypes of this malignancy indicated a time-dependent and very similar attenuation of cell growth after CAP treatment. A significant cellular growth reduction of 50% could be achieved with a single CAP treatment of 10 s [23]. Subsequent molecular analysis pointed to a CAP induced activation of apoptotic mechanisms. Both, the induction as well as the phospho-activation of the oncogenic p53 protein were detected, followed by nuclear pyknosis, the apoptosis-specific shrinking, and degradation of the nucleus [23]. In comparison of SaOS-2 osteosarcoma cells and human mesenchymal stem cells, the cancer cells died exclusively by induction of apoptosis while non-malignant mesenchymal cells remain fully viable and unaffected after CAP treatment [24]. Moreover, and due to the composition of CAP containing charged and highly reactive particles, the involvement of the cellular redox machinery has been shown in osteosarcoma cells. CAP’s cellular effects could be neutralized by the supplementation of N-acetylcysteine, which could be metabolized to the cellular antioxidant glutathione [25]. Furthermore, CAP treatment led to an inactivation of peroxiredoxin-1 and peroxiredoxin-2, but not the mitochondria-specific isoform peroxiredoxin-3 [25]. Peroxiredoxines are not only involved in cellular redox signalling, but also in apoptosis regulation [26].

## Bladder cancer

There are two studies evaluating CAP effects on the SCaBER bladder cancer cell culture model demonstrating anti-proliferative and pro-apoptotic properties of CAP [27], [28]. By transcriptomic profiling of CAP treated cells applying a genome-wide DNA array, Keidar et al. identified 264 genes whose expression rates were significantly modulated after CAP treatment [28]. The genes encoding proteins were primarily involved in cell adhesion, cell growth, and cell death. A subsequent ingenuity pathway analysis (IPA) enabled the prediction of involved signal transduction cascades. Particularly, signal cascades of cell development, cell death, cell motility, and inflammation were affected. All of these regulatory mechanisms are highly engaged in cancer initiation and progression.

## Prostate cancer

Similar results were found with prostate cancer cells, promising successful CAP application in prostate cancer therapy [29]. Already a single CAP treatment of 10 s exhibited antiproliferative effects in prostate cancer cells LNCaP and PC-3 incubated over 120 h [30], which was confirmed with DU-145 prostate cancer cells exposed to CAP [31]. The observed effects were comparable to those in the presence of 10 µM docetaxel, a taxane compound clinically used in advanced prostate cancer therapy. On the level of molecular cell biology several factors have been identified interfering with CAP efficacy. The cell cycle regulator p53 as well as the pro-apoptotic factors BAX and p21 were induced following CAP treatment and, vice versa, the expression of the anti-apoptotic protein survivin was attenuated [30]. Consequently, the induction of apoptotic pathways led to the activation of caspases [32] and subsequent induction of DNA strand breaks [33], [34] and nuclear degradation [34]. Similar to CAP-induced effects in osteosarcoma cells, cell response of CAP-treated prostate cancer cells included the activation of redox signalling cascades [35], [36].

## Conclusion

Plasma oncology opens up completely new opportunities for oncological surgery. As an additional option, the intraoperative direct CAP treatment of malignant tissue as well as CAP treatment of wound edges after resection may become a promising option in cancer therapy. Notably, CAP efficacy is not limited to specific antiproliferation, but also to further beneficial effects including microbial decontamination, immunostimulation, and promotion of wound healing and scarring.

## Notes

### Competing interests

The authors declare that they have no competing interests.
